# Platelet-derived growth factor modulates rat vascular smooth muscle cell responses on laminin-5 via mitogen-activated protein kinase-sensitive pathways

**DOI:** 10.1186/1478-811X-3-2

**Published:** 2005-01-31

**Authors:** Karl Kingsley, George E Plopper

**Affiliations:** 1 Department of Biomedical Sciences, University of Nevada, Las Vegas, School of Dental Medicine, 1001 Shadow Lane B-234, Las Vegas, Nevada, 89106-4124, USA; 2 Department of Biology, Rensselear Polytechnic Institute, 110 8^th ^Street, Troy, New York, 12180-3596, USA; 3 (previous institutional affiliation) Department of Biological Sciences, University of Nevada, Las Vegas, 4505 Maryland Parkway, Box 454004, Las Vegas, Nevada, 89154-4004, USA

## Abstract

**Background:**

A treatment to remove vascular blockages, angioplasty, can cause damage to the vessel wall and a subsequent abnormal wound healing response, known as restenosis. Vascular smooth muscle cells (VSMC) lining the vessel wall respond to growth factors and other stimuli released by injured cells. However, the extracellular matrix (ECM) may differentially modulate VSMC responses to these growth factors, such as proliferation, migration and adhesion. Our previous reports of low-level expression of one ECM molecule, laminin-5, in normal and injured vessels suggest that laminin-5, in addition to growth factors, may mediate VSMC response following vascular injury. To elucidate VSMC response on laminin-5 we investigated-the role of platelet-derived growth factor (PDGF-BB) in activating the mitogen-activated protein kinase (MAPK) signaling cascade as a possible link between growth-factor initiated phenotypic changes *in vitro *and the ECM.

**Results:**

Using a system of *in vitro *assays we assessed rat vascular smooth muscle cell (rVSMC) responses plated on laminin-5 to the addition of exogenous, soluble PDGF-BB. Our results indicate that although laminin-5 induces haptotactic migration of rVSMC, the addition of PDGF-BB significantly increases rVSMC migration on laminin-5, which is inhibited in a dose-dependent manner by the MAPK inhibitor, PD98059, and transforming growth factor (TGF-β1). In addition, PDGF-BB greatly reduces rVSMC adhesion to laminin-5, an effect that is reversible by MAPK inhibition or the addition of TGF-β1. In addition, this reduction in adhesion is less significant on another ECM substrate, fibronectin and is reversible using TGF-β1 but not MAPK inhibition. PDGF-BB also strongly increased rVSMC proliferation on laminin-5, but had no effect on rVSMC plated on fibronectin. Finally, plating rVSMC on laminin-5 did not induce an increase in MAPK activation, while plating on fibronectin or the addition of soluble PDGF-BB did.

**Conclusion:**

These results suggest that rVSMC binding to laminin-5 activates integrin-dependent intracellular signaling cascades that are different from those of fibronectin or PDGF-BB, causing rVSMC to respond more acutely to the inhibition of MAPK. In contrast, our results suggest that fibronectin and PDGF-BB may activate parallel, reinforcing intracellular signaling cascades that converge in the activation of MAPK and are therefore less sensitive to MAPK inhibition. These results suggest a partial mechanism to explain the regulation of rVSMC behaviors, including migration, adhesion, and proliferation that may be responsible for the progression of restenosis.

## Background

Angioplasty is a procedure designed to treat vascular stenosis, blockage(s), or atherosclerotic lesions, but it may also, simultaneously, cause damage to the integrity of the blood vessel wall. Restenosis is the subsequent narrowing and occlusion of the blood vessel in response to the injury or damage sustained during angioplastic procedures such as balloon dilation [1]. During restenosis, vascular smooth muscle cells (VSMC) from the injured blood vessel wall migrate into the lumen of the vessel, creating a new or neointima. The subsequent proliferation of these neointimal VSMC can lead to a thickening of this neointimal layer and re-occlusion of the vessel [1].

The characteristic response of VSMC, endothelial cells, platelets, and macrophages at the site of injury is the release of specific soluble growth factors which include platelet-derived growth factor (PDGF), transforming growth factor (TGF), basic fibroblast growth factor (bFGF), and epidermal growth factor (EGF) [2-4]. VSMC of the vessel wall respond to these factors by secreting proteolytic matrix metalloproteinases that degrade the extracellular matrix (ECM) and stimulate deposition of new ECM proteins such as collagen, elastin, fibronectin, and laminin in the neointima [5-7]. These ECM modulate VSMC integrin-dependent behaviors such as transluminal migration, adhesion, and proliferation [8-10].

To date, the precise molecular mechanisms that link growth factor-initiated intracellular signaling to ECM-mediated adhesion, migration, and proliferation of VSMC are still unknown. Our previous reports of low-level laminin-5 expression in the intima of normal vasculature and an increased expression of laminin-5 in the neointima of injured vessels suggest that laminin-5, in addition to PDGF and TGF, may mediate VSMC responsiveness following vascular injury [11-13].

To further elucidate VSMC response to growth factors and the intracellular signaling cascades that may be linked to ECM-mediated adhesion, we used *in vitro *assays to study the role of laminin-5 in modulating these behaviors in rat vascular smooth muscle cells (rVSMC). We report here that PDGF induces differential responses in rVSMC behaviors on laminin-5, but not on fibronectin. In addition, we find that the PDGF-induced responses on laminin-5 are inhibited in a dose-dependent manner by an inhibitor of the mitogen-activated protein kinase (MAPK) pathway, PD98059, but not on fibronectin.

These differences in MAPK-sensitive rVSMC responses *in vitro *may be the result of different signaling pathways that are initiated by integrin-mediated adhesion to laminin-5. We suggest that rVSMC binding to laminin-5 may initially activate a MAPK-independent signaling cascade that may make these cells more responsive to MAPK inhibition. In contrast, rVSMC binding to fibronectin may activate a signaling pathway shared by PDGF that ultimately converges in MAPK activation. These results suggest that a complex interaction of ECM and growth factors may closely regulate VSMC behavior following vascular injury and these studies may directly identify define molecular targets that may reduce the incidence of restenosis following angioplasty.

## Results

### Migration

We have previously reported that laminin-5 expression by rat vascular smooth muscle cells (rVSMC) is upregulated by platelet-derived growth factor (PDGF-BB) and that laminin-5 specifically enhances PDGF-BB-stimulated rVSMC migration [12]. In addition, our previous results suggested that PD98059 (a specific inhibitor of MEK and a general inhibitor of the mitogen-activated protein kinase ERK1/2 pathway) blocked PDGF-BB-stimulated migration on laminin-5 [13]. The current study characterizes this synergistic relationship between PDGF-BB and laminin-5 in the modulation of rVSMC cell behaviors, including migration.

**Figure 1:**
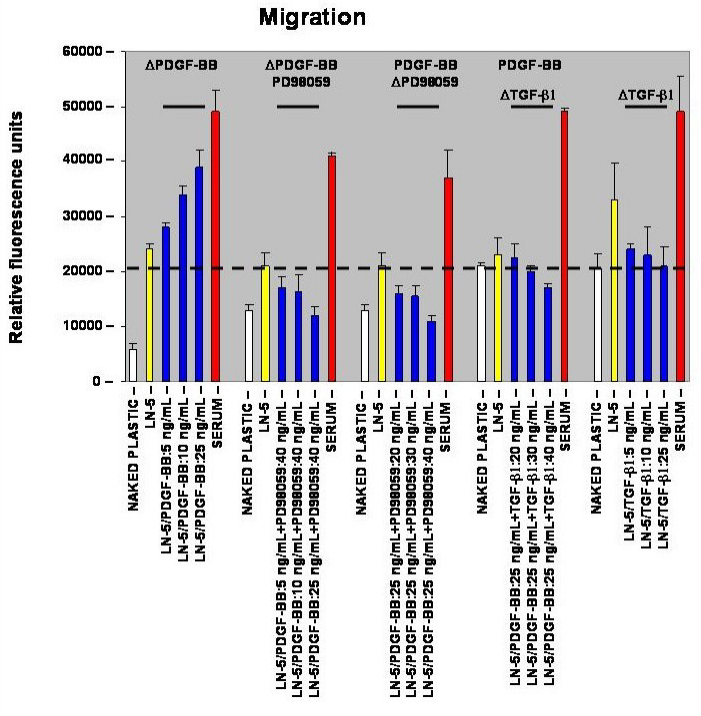
**PDGF-BB increased rVSMC migration on laminin-5 *in vitro*. **The addition of PDGF-BB increased rVSMC migration on laminin-5, in a dose-dependent manner, over rVSMC haptotactic migration induced by the presence of laminin-5. rVSMC migration on laminin-5 was inhibited by both PD98059 (MEK1 inhibitor) and TGF-β1, in a dose-dependent manner.

The level of rVSMC migration was observed over 18 hours in transwell migration filters in the presence or absence of laminin-5, with and without PDGF-BB or serum. Maximal cell migration (chemotaxis) was obtained using cell migration media containing ten (10) percent fetal calf serum (FCS). Similar to our previous reports, laminin-5-coated wells induced a greater than four-fold increase in rVSMC migration over that measured in naked plastic controls in three independent experiments as shown in Figure 1 (n = 24, p < 0.05). Furthermore, PDGF-BB, tested over a biologically relevant range of 5 – 50 ng/mL, stimulated a dose-dependent increase in rVSMC migration on laminin-5 (between 5 – 25 ng/mL), increasing migration fifteen (15) to sixty (60) percent over laminin-5-stimulated migration alone (n = 24, p < 0.005). This PDGF-BB-stimulated increase in migration peaked at 25 ng/mL and did not significantly increase over the range of 25 – 50 ng/mL (data not shown).

The mitogen-activated protein kinase (MAPK) pathway is known to be activated by PDGF-BB-stimulation in VSMC [14]. To explore the mechanism of increased rVSMC migration on laminin-5 via PDGF-BB-stimulation, we used PD98059 which is a specific inhibitor of MEK1 and MEK2, and a general inhibitor of MAPK activation. The addition of PD98059 in concentrations between 10 – 50 ng/mL reduced PDGF-BB-stimulated migration on laminin-5 (25 ng/mL) in a dose-dependent manner, reaching a maximal reduction of sixty (60) to seventy (70) percent over the range of 20 – 40 ng/mL, as shown in Figure 1 (n = 24, p < 0.005). This reduction in PDGF-BB-stimulated migration on laminin-5 was maintained at all concentrations of PDGF-BB tested (5 – 25 ng/mL).

To determine if this modulation is restricted to MEK/MAPK-inhibition, we tested the effects of adding exogenous transforming growth factor (TGF-β1) for its ability to modulate the PDGF-stimulated increase in rVSMC migration on laminin-5. Our results indicated that the addition of TGF-β1 was able to reduce laminin-5-stimulated rVSMC migration to levels approximating the levels observed in naked plastic controls over all tested ranges (5 – 50 ng/mL), although the most statistically distinct reduction was found over the range of 5 – 25 ng/mL, as shown in Figure 1. In addition, TGF-β1 was able to reduce maximal PDGF-BB-stimulated migration of rVSMC (25 ng/mL) on laminin-5 by approximately fifty (50) to sixty (60) to percent, over the range of concentrations from 20 to 40 ng/mL.

### Adhesion

**Figure 2:**
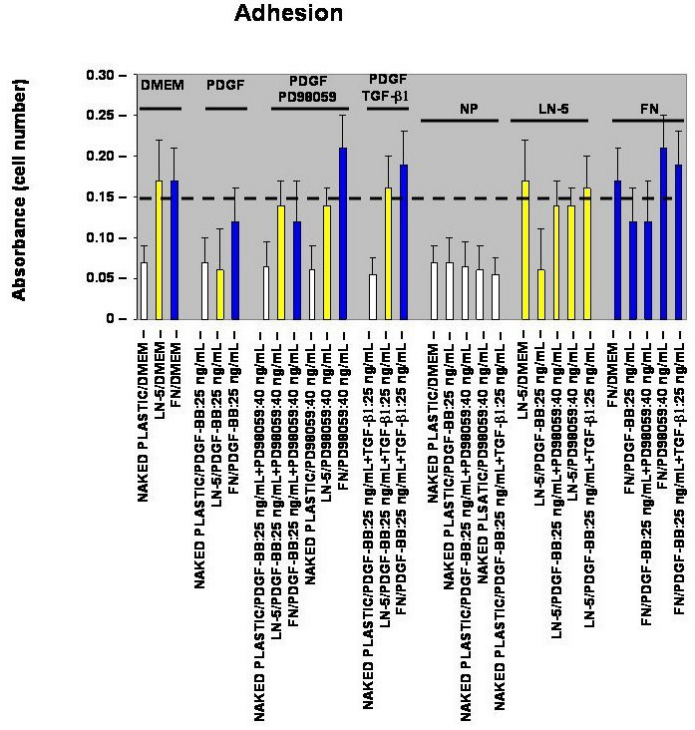
**PDGF-BB reduces rVSMC adhesion on laminin-5 adhesion *in vitro***. The presence of laminin-5 supported greater adhesion of rVSMC over naked plastic, and this increase in adhesion was reduced by the addition of PDGF-BB. Inhibition of MEK1, using PD98059 restored rVSMC adhesion to laminin-5 in the presence of PDGF-BB. Although fibronectin also supported rVSMC adhesion, the effect of adding PDGF-BB was less pronounced and was not restored using PD98059. The addition of TGF-β1, however, completely restored rVSMC adhesion to both fibronectin and laminin-5 in the presence of PDGF-BB.

To determine whether or not the PDGF-BB-stimulated increase in rVSMC migration correlates with a reduction in rVSMC adhesion to laminin-5, thirty-minute *in vitro *adhesion assays were performed. In the absence of exogenous growth factor stimulation, laminin-5- and fibronectin-coated wells (20 μg/mL) sustained an approximate two and a half-fold increase in rVSMC adhesion compared with negative controls, as shown in Figure 2 (n = 24, p < 0.05). The addition of PDGF-BB, at the maximal migration-stimulating dose of 25 ng/mL, decreased the adhesion of rVSMC on laminin-5 by more than sixty-five (65) percent (n = 24, p < 0.005). The addition of exogenous PDGF-BB (25 ng/mL), however, decreased rVSMC adhesion on fibronectin by less than thirty (30) percent.

To investigate if the relationship between the PDGF-BB-stimulated increase in migration and corresponding decrease in adhesion of rVSMC on laminin-5 may be related to MAPK activation, cells were pre-treated with PD98059 (40 ng/mL) for twenty (20) minutes prior to assay and the adhesion media was supplemented with PD98059 (40 ng/mL). The addition of exogenous PD98059 (40 ng/mL) restored the PDGF-BB-stimulated reduction (25 ng/mL PDGF-BB) in rVSMC adhesion to laminin-5, to approximately eighty-five (85) percent of laminin-5 controls (n = 24, p < 0.05). The addition of PD98059, however, did not restore the thirty (30) percent PDGF-BB-stimulated reduction in rVSMC adhesion on fibronectin.

To determine if this modulation is restricted to MEK/MAPK-inhibition, we tested the effects of adding exogenous transforming growth factor (TGF-β1). Our results indicated that the addition of TGF-β1 (25 ng/mL) was able to restore the PDGF-BB-stimulated reduction in rVSMC adhesion on laminin-5 by roughly the same level as PD98059, eighty two (82) percent versus eighty five (85) percent, respectively. In contrast to PD98059, the addition of TGF-β1 (25 ng/mL) was sufficient to restore the PDGF-BB-stimulated reduction in rVSMC adhesion on fibronectin.

### Proliferation

Based upon our observations of rVSMC migration and adhesion, we performed *in vitro *proliferation assays to determine the relative effects of the extracellular matrix (ECM) and exogenous growth factors described above. First, to test the effects of the ECM substrate on rVSMC proliferation in the absence of exogenous growth factors, quiescent cells were plated in cell-culture plates either coated with or without laminin-5 or fibronectin, in serum-free Dulbecco's Modified Eagle's Medium (DMEM) for one (1) to six (6) days.

**Figure 3:**
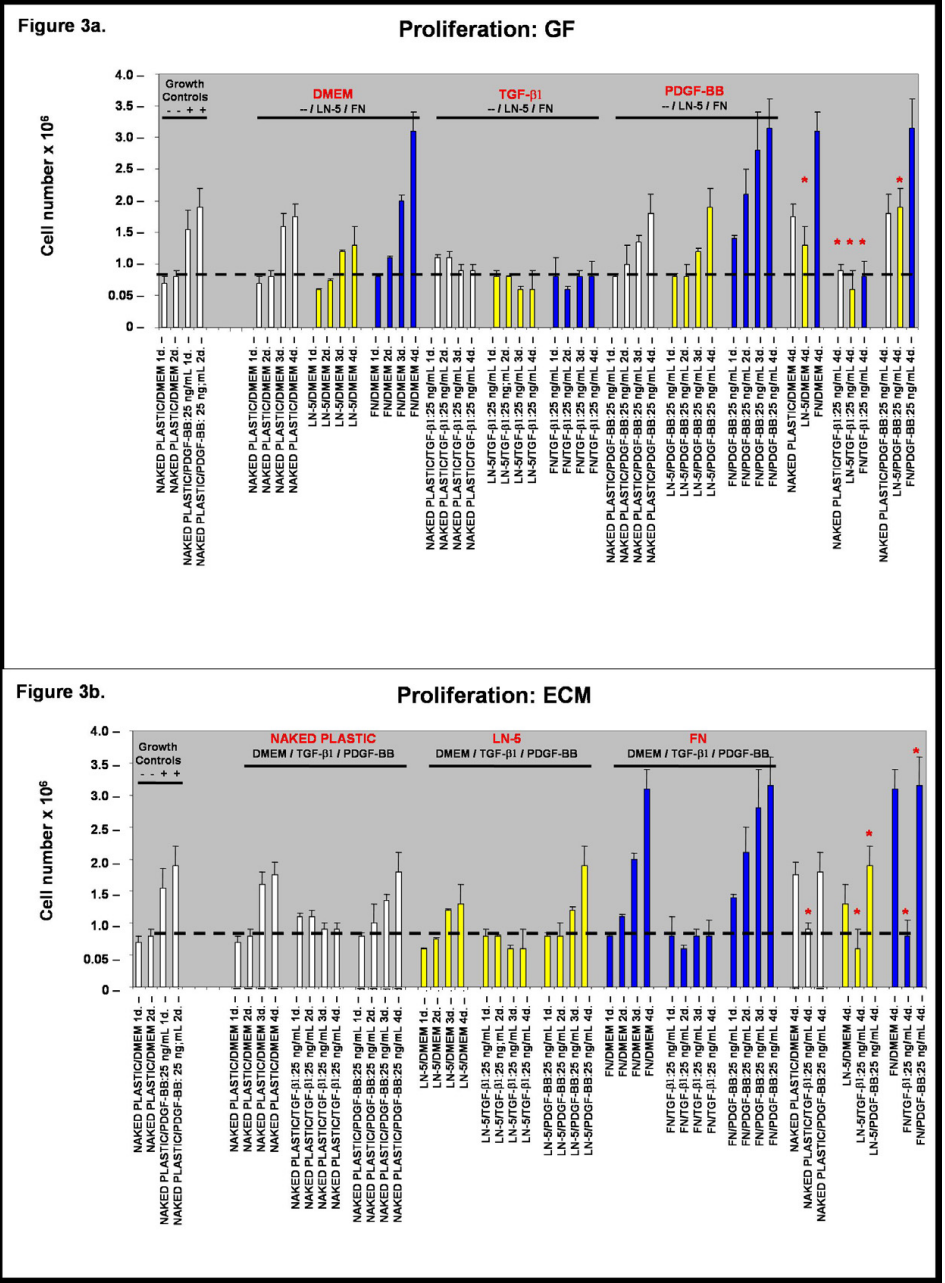
**The effect of growth factors on rVSMC proliferation *in vitro*. **The addition of PDGF-BB stimulated proliferation of rVSMC on laminin-5 and to some extent on naked plastic, but not on fibronectin. The presence of fibronectin alone was able to stimulate proliferation of rVSMC. The proliferative response of rVSMC to presence of laminin-5 with PDGF-BB or to fibronectin was suppressed by the addition of TGF-β1. **Figure 3b** The ECM induces differential rVSMC proliferation responses *in vitro*. The plating of rVSMC on fibronectin, but not laminin, induced increases in proliferation over four days. The addition of PDGF-BB, however, increased rVSMC migration on laminin-5, but not on fibronectin. The addition of TGF-β1 was sufficient to suppress rVSMC proliferation on both laminin-5 and fibronectin.

To induce quiescence, rVSMC were incubated for forty eight (48) hours without mitogen (0% FCS, FBS) at 37°C and quiescence was verified by proliferation controls, cultured for forty eight (48) hours at 37°C with mitogen stimulus as shown in Figure 3a and 3b. Previous studies with VSMC report that greater than 95% of cells incubated in low-serum media (0.4% FCS or less) were arrested in G_0_(G_1_) between forty eight (48) and seventy two (72) hours as determined by flow cytometry and determination of [3H] thymidine-labeled nuclei [15].

Our results suggest that culture of rVSMC plated on exogenous laminin-5 (coated at a concentration of 20 μg/mL) did not significantly increase cellular proliferation compared with naked plastic controls over a period of four (4) days, as shown in Figure 3a (n = 24, p < 0.02). Culturing of rVSMC plated on exogenous fibronectin (coated at a concentration of 20 μg/mL) however, did significantly increase cellular proliferation over a period of four (4) days by nearly two-fold, as shown in Figures 3a and 3b.

Next, to test the modulating effects of exogenous growth factors on rVSMC proliferation when cultured on these ECM substrates, quiescent cells were plated in cell-culture plates either naked or coated with laminin-5 or fibronectin, in the presence of TGF-β1 or PDGF-BB, both at a concentration of 25 ng/mL, from one (1) to six (6) days. Our results indicated that the addition of exogenous TGF-β1 (25 ng/mL) to the cell culture medium was sufficient to induce a suppressing effect on rVSMC proliferation on naked plastic, as well as laminin-5 and fibronectin, to levels approximating the quiescent growth controls.

The addition of PDGF-BB was sufficient to induce an increase in rVSMC proliferation on laminin-5 of nearly one-half (46%) over laminin-5 DMEM (no serum, no mitogen) controls (n = 24, p < 0.01). However, our results indicate that the addition of exogenous PDGF-BB (25 ng/mL) did not have a statistically significant effect on cells plated on fibronectin.

### Mitogen Activated Protein Kinase (MAPK) Western Blot

**Figure 4:**
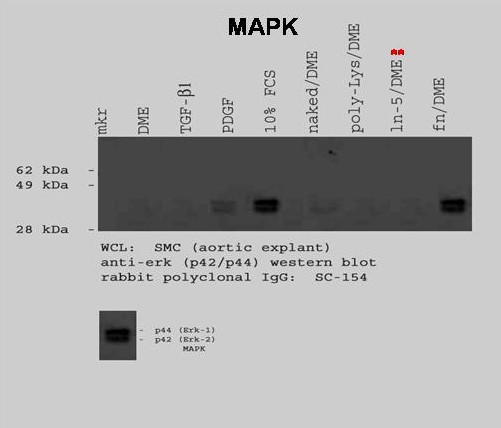
**The MAPK pathway in rVSMC is activated by different stimuli**. The addition of FCS or PDGF-BB or the plating of rVSMC on fibronectin was sufficient to induce measurable increases in p44/p42 activation over 30 minutes. However, the addition of TGF-β1 or the plating of rVSMC on laminin-5 was not sufficient to induce MAPK activation.

To examine MAPK activation, rVSMC were plated onto laminin-5- or fibronectin-coated plates, then lysed after thirty minutes and prepared for immunoblotting. Our results indicated that laminin-5 did not induce a detectable increase in MAPK activation over a thirty (30) minute time interval, as shown in Figure 4. rVSMC plated on fibronectin did exhibit a significant increase in MAPK levels after thirty (30) minutes.

The addition of growth factors, such as PDGF-BB and TGF-β1, have been associated with increases in p44/p42 (ERK1/2) or MAPK activation [16-18]. To determine the effects of PDGF-BB and TGF-β1 on rVSMC, subconfluent cell cultures were pre-treated with FCS (10%), PDGF-BB (25 ng/mL) or TGF-β1 (25 ng/mL) for thirty (30) minutes, then lysed and prepared for immunoblotting. p44/p42 phosphorylation levels were not detectable in DMEM-treated control cells, as shown in Figure 4. However, the addition of PDGF-BB, but not TGF-β1, was sufficient to induce measurable increases in MAPK activation levels.

## Discussion

PDGF-BB is an *in vitro *VSMC mitogen and may be responsible for initiating the phenotypic changes in VSMC migration and proliferation during restenosis *in vivo *[19,20]. Our recent reports of low-level laminin-5 expression in the intima of normal arteries and increased expression in the neointima of injured arteries suggest that laminin-5, in conjunction with soluble growth factors and mitogens, may determine VSMC phenotype during restenosis [11-13].

The current study augments the body of evidence that suggests VSMC growth is influenced by an ECM-VSMC interaction [21] and that VSMC proliferation in different species respond differently to growth factor stimulus [15]. More specifically, this study explores evidence that laminin-5 and PDGF-BB may have combined and synergistic effects in determining rVSMC phenotype *in vitro *[6,8,10]. More specifically, our studies suggest that PDGF-BB strongly influences rVSMC behaviors such as migration. In addition, PDGF-BB significantly alters other cellular behaviors such as adhesion and proliferation on laminin-5, but only to a lesser extent on fibronectin.

Because PDGF-BB stimulation increases the overall levels of intracellular MAPK, as well as MAPK phosphorylation, we sought to explore this signaling cascade to determine its role in modulating these rVSMC behaviors on laminin-5 [22]. Specifically, the ERK1/2 form of MAPK mediates signaling by PDGF-AA, PDGF-AB, and PDGF-BB in VSMC, as well as signaling through laminin-5 binding integrins, and is therefore the most likely signaling molecule to modulate these cellular behaviors [23-25]. Our results suggest that rVSMC behaviors *in vitro*, driven by PDGF-BB responsiveness, can be blocked by MAPK inhibition (via MEK1 inhibitor: PD98059) on laminin-5, but not on fibronectin.

An additional signaling regulator, TGF-β1, has been implicated in the negative regulation and decreased rate of proliferation of VSMC stimulated with serum or PDGF [26-28]. Our results from this study indicate that TGF-β1, unlike the MEK1-inhibitor PD98059, was sufficient to block rVSMC behaviors on both laminin-5 and fibronectin. Specifically, the addition of TGF-β1 was able to reduce PDGF-BB-stimulated migration and proliferation of rVSMC on both ECM substrates and was also able to restore PDGF-BB-stimulated reductions in VSMC adhesion on these ECM.

Several lines of evidence now suggest that the anti-mitogenic effects of TGF-β1 may be dissociated from inhibition of ERK1/2 signaling pathways [17,18]. These reports suggest that TGF-β1 inhibition of PDGF-BB may be temporally independent of other early signaling pathways, such as MAPK, and is more likely to block VSMC behaviors, such as proliferation, by inhibiting events later in the G_1 _phase of mitosis.

Although our previous reports linked ERK1/2 to rVSMC adhesion and migration, these studies did not examine the possibility for differential signaling initiated by rVSMC binding to laminin-5 or fibronectin [12,13]. Expanding our original analysis of growth factor stimulation of MAPK to include ECM binding reveals that integrin binding of rVSMC to fibronectin strongly increases detectable MAPK activation levels, as does FCS and PDGF-BB stimulation, whereas binding to laminin-5 does not. These differences may help to explain the differing effects on cellular behaviors of binding to these ECM ligands, as fibronectin and PDGF-BB may act in unison to activate intracellular signaling cascades that converge in MAPK activation, while laminin-5 may not.

## Conclusions

**Figure 5:**
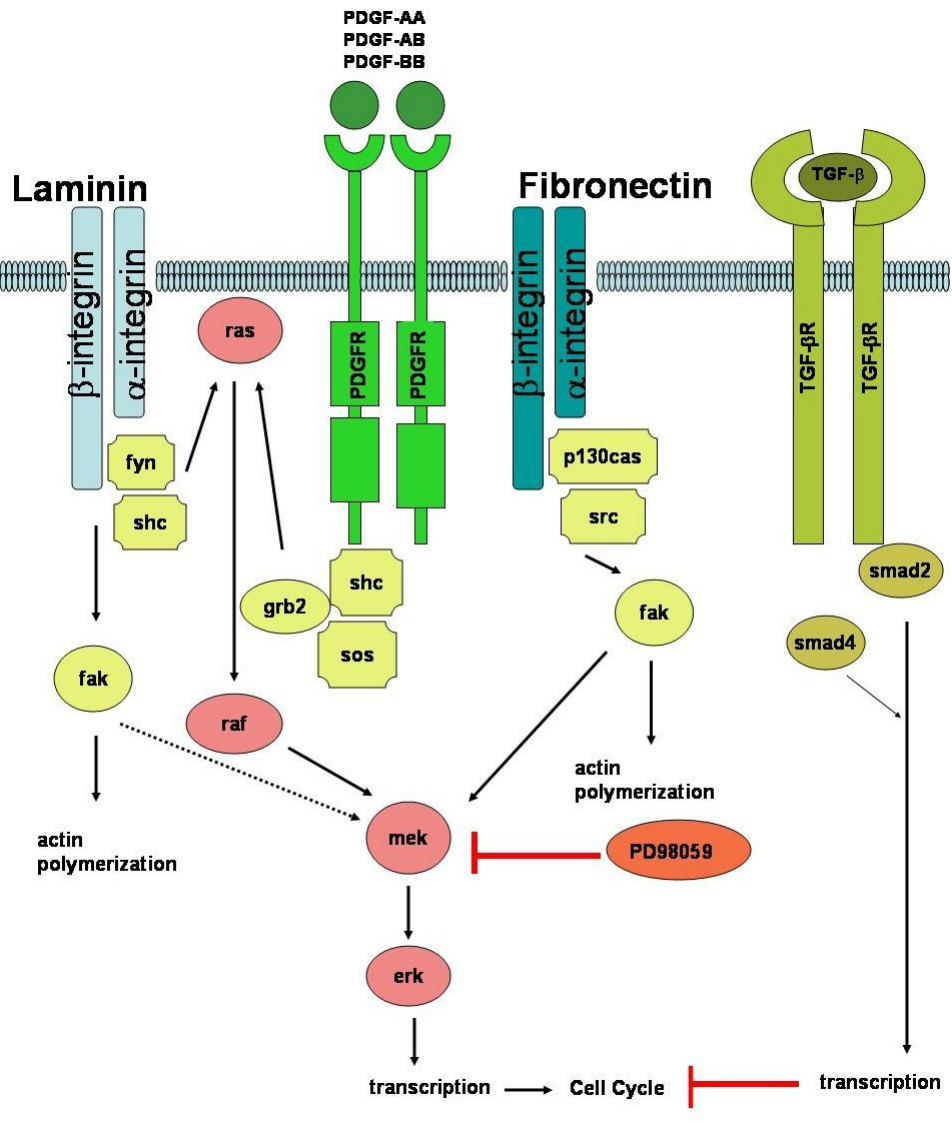
**Integrins and growth factors activate intracellular signaling cascades in rVSMC. **Intracellular signaling pathways that converge through MEK and ERK activation, may be initiated in rVSMC by the addition of PDGF-BB, as well as binding to laminin-5 or fibronectin. The MEK1 inhibitor PD98059 blocks MEK1 activation and alters rVSMC responses to PDGF-BB on laminin-5, but not fibronectin. The addition of TGF-β1, which may block later events in the cell cycle, is sufficient to block PDGF-BB induced responses of rVSMC on both laminin-5 and fibronectin.

Our results indicate that laminin-5 activates different intracellular signaling pathways from those of fibronectin and PDGF-BB in rVSMC and that binding to laminin-5 may modulate rVSMC behaviors that are distinctive from those modulated by fibronectin. Although binding of rVSMC to laminin-5 may not cause an initial increase in MAPK activation levels or proliferation, laminin-5 can augment PDGF-BB-stimulated proliferation and migration of rVSMC *in vitro*. Based upon these findings, we postulate that PDGF-BB and laminin-5 binding may initially activate different intracellular signaling cascades, causing rVSMC to be more responsive to the inhibition of MEK1 and MAPK on laminin-5 than those activated on fibronectin, as outlined in Figure 5.

In contrast to laminin-5, fibonectin and PDGF-BB may have parallel, reinforcing roles in MAPK activation. Our analysis of the effects of TGF-β1 demonstrated that TGF-β1 does not strongly activate MAPK in rVSMC, but rather strongly inhibits the effects of fibronectin and PDGF-BB-stimulation on laminin-5. Our results support the previous findings that TGF-β1 may inhibit mitogenesis and other VSMC behaviors via mechanisms independent of MAPK activation.

These results suggest that a clear understanding of the roles and contributions of each ECM may provide new insights into the mechanisms of regulating rVSMC cell behaviors, including migration, adhesion, and proliferation. Further analysis of the events that trigger and sustain the underlying cellular mechanisms of rVSMC behaviors may help aid in the design of more effective therapies for the treatment of restenosis.

## Methods

### Cell culture

Cells were maintained in 100 mm × 20 mm Corning tissue-culture dishes (Plainfield, NJ) at 37°C and 5% CO_2 _in humidified chambers. Cells were maintained in DMEM High Glucose, supplemented with 10% fetal bovine serum and 1% L-glutamine (29.2 mg/mL), penicillin G (10,000 U/mL), and streptomycin sulfate (10,000 mcg/mL) (GPS) from Irvine Scientific (Santa Ana, CA). Rat aortic smooth muscle cell explants were a gift from RC Smith and were isolated and passaged as previously described [29]. Although greater than 95% quiescence, G_0_(G_1_) arrest can routinely be induced by incubation of cells for 72 h. in low-mitogen (0.5% FBS) medium [15,30], these authors suggest that incubation of VSMC for 48 h. without mitogen (0% FBS) is sufficient to induce quiescence. This was verified by proliferation control cells, cultured for 48 h. at 37°C with and without mitogen stimulus.

### Migration assays

Cell migration assays were performed in Costar transwell filter plates either coated with purified matrix (laminin-5 or fibronectin) at a protein concentration of 20 μg/mL for one hour (60 min.) at room temperature, 25°C, and washed twice with phosphate-buffered saline 0.2% Tween-20 and 5% skim milk (PBST) prior to assay as previously described [31,32]. Cells were seeded at a concentration of 1.2 × 10^5 ^in each of 96-transwell chamber filters (100 μL of 1.2 × 10^6 ^cells/mL solution) with and without ECM in the presence or absence of PDGF-BB at the indicated concentrations (5–25 ng/mL) and allowed to migrate for 18 hours at 37°C. Where applicable, the medium was supplemented with PD98059 (MEK1-inhibitor) at the indicated concentration. Cells were counted at the end of an 18-hour interval as indicated, quantified with the following modification. 30 minutes prior to measuring migration, 5 μM calcein AM from Molecular Probes (Eugene, OR) was added to the migration wells at 37°C. To quantitate migration, cells were removed from the top of the filter with cotton-tipped applicators and fluorescence of the incorporated calcein was measured from the bottom of the filter with a fluorescence plate reader. Relative fluorescence values for each experimental condition are expressed relative to controls and untreated samples.

### Adhesion assays

Cell adhesion assays were performed as previously described [31,32] using Costar 96-well cell culture cluster plates, coated with either laminin-5 or fibronectin solution at a protein concentration of 20 μg/mL for 1 hour (60 min.) at room temperature, 25°C. Wells were then washed twice with PBST prior to assay. Cells were seeded at a concentration of 1.2 × 10^5 ^in each of 96-transwell chamber filters (100 μL of 1.2 × 10^6^cells/mL solution) with and without ECM-coating (described above) in the presence or absence of PDGF-BB (25 ng/mL), TGF-β1 (25 ng/mL), or both, and allowed to attach for 30 minutes at 37°C. Where applicable, cells were first incubated for 20 minutes with PD98059 (40 ng/mL), a MEK1 inhibitor from New England Biolabs (Beverly, MA) at 37°C and the adhesion assay culture medium was supplemented with PD98059 at 40 ng/mL. Following adhesion, non-adherent cells were removed by suspending plates upside down in a rotating tank of PBS for 10 minutes at room temperature, 25°C. Adherent cells were then fixed and stained and the relative absorbance was measured using a TECAN-SPECTRAFluor spectrophotometer (TECAN, Durham, NC) at 595 nm.

### Proliferation assays

Tissue culture plates were coated with purified fibronectin from Calbiochem (La Jolla, CA) or laminin-5 from Demos (La Jolla, CA) at a 20 μg/mL protein concentration for 1 hour (60 min.) at room temperature, 25°C as previously described [31]. Cells were seeded at a concentration of 1.2 × 10^5 ^in 100 mm^2 ^cell culture plates with and without fibronectin or laminin-5 and allowed to attach overnight (12 h.) at 37°C. Cells were then starved in serum-free DMEM for 48 hours to induce quiescence at 37°C, as outlined in Cell Culture Methods below. The medium was then replaced with fresh medium containing 25 ng/mL of TGF-β1 or PDGF-BB obtained from Calbiochem (La Jolla, CA) and incubated at 37°C. Cells were removed from culture wells with trypsin/EDTA and counted using trypan blue stain from Gibco Life Technologies (Rockville, MD) and a VWR Scientific Counting Chamber (Plainfield, NJ) at 24 hour intervals, from 1 – 6 days.

### Western Blot analysis

Quiescent VSMC were pre-treated with culture medium containing 10% FCS, PDGF-BB (25 ng/mL), TGF-β1 (25 ng/mL), or plated on laminin-5- or fibronectin-coated tissue culture plates with DMEM for 30 minutes at 37°C. Subconfluent cell cultures were then lysed using ice-cold, 1X lysis buffer (50 mM Tris-HCl, 150 mM NaCl, 5 mM EDTA, 1.0% Triton X-100, pH 7.5) and boiled in SDS Boiling Buffer prior to analysis as previously described [33]. Proteins were separated by 7.5% SDS-PAGE and transferred to Immobilon-P transfer membranes from Millipore (Bedford, MA), incubated with primary antibody (1:200 dilution at 4°C overnight, 12 h.), secondary antibody (1:2000 dilution at 25°C for 1 h.), and then exposed to NEN CDP-Star chemiluminescence reagent (5–7 min.) and developed on Kodak X-OMAT LS scientific imaging film for subsequent analysis. Antibodies used for this analysis were anti-phospho ERK (p42/p44) rabbit polyclonal IgG primary antibody and goat anti-rabbit IgG-AP secondary antibody from Santa Cruz Biotechnology (Santa Cruz, CA).

### Statistics

The differences between untreated and treated cell populations were measured using a *t *distribution. All samples were measured using two-tailed *t *tests as departure from normality can make more of a difference in a one-tailed than in a two-tailed *t *test. So long as the sample size is even moderate (>20) for each group, quite severe departures from normality make little practical difference in the conclusions reached from these analyses [34].

## Competing Interests

The author(s) declare that they have no competing interests.

## Authors' contributions

KK carried out the migration, adhesion, and proliferation assays, the Western Blot analysis and assisted with experimental design. GEP conceived, monitored, and coordinated the experimental design. Both KK and GEP contributed equally to the writing of this manuscript.
